# Vaginal *Prevotella timonensis* Bacteria Enhance HIV‐1 Uptake and Differentially Affect Transmission by Distinct Primary Dendritic Cell Subsets

**DOI:** 10.1002/eji.202451192

**Published:** 2025-03-12

**Authors:** Marleen Y. van Smoorenburg, Ester B. M. Remmerswaal, Celia Segui‐Perez, John L. van Hamme, Karin Strijbis, Teunis B. H. Geijtenbeek

**Affiliations:** ^1^ Department of Experimental Immunology Amsterdam UMC location University of Amsterdam Amsterdam The Netherlands; ^2^ Amsterdam institute for Immunology and Infectious Diseases Infectious Diseases Amsterdam The Netherlands; ^3^ Department of Biomolecular Health Sciences Division Infectious Diseases and Immunology Faculty of Veterinary Medicine University of Utrecht Utrecht The Netherlands

**Keywords:** dendritic cells (DCs), HIV‐1 susceptibility, *Prevotella timonensis*, primary CD1c^+^ DCs, transmission, vaginal dysbiosis, vaginal microbiome, viral uptake

## Abstract

Young females are at high risk of acquiring HIV‐1 infections and an imbalance in the vaginal microbiome enhances susceptibility to HIV‐1 infection. More insights into the underlying mechanisms could open up new strategies to prevent HIV‐1 acquisition and dissemination. Here, we investigated the effect of anaerobic bacteria associated with bacterial vaginosis (BV) on HIV‐1 transmission by two distinct dendritic cell (DC) subsets, that is, inflammatory monocyte‐derived DCs (moDCs) and primary CD1c^+^ DCs. Notably, in contrast to other BV‐associated microbiota, *Prevotella timonensis* enhanced uptake of HIV‐1 by both moDCs and CD1c^+^ DCs and the increased uptake was independent of cellular HIV‐1 (co‐)receptors. Imaging flow cytometry analyses showed that HIV‐1 did not co‐localise with *P. timonensis* but was internalized into tetraspanin‐positive compartments known to be involved in HIV‐1 transmission. *P. timonensis* bacteria enhanced HIV‐1 transmission by CD1c^+^ DCs, but not by moDCs, and the enhanced transmission was independent of viral infection. Our study strongly suggests that mucosal DC subsets have distinct functions in BV‐associated HIV‐1 susceptibility, and underscores the importance of early diagnosis and targeted treatment of vaginal dysbiosis to reduce the risk of HIV‐1 acquisition.

## Graphical abstract

The bacterium *Prevotella timonensis* found in vaginal dysbiosis enhances the uptake of HIV‐1 by primary dendritic cells. Virus is internalised into tetraspanin‐rich compartments, which leads to efficient HIV‐1 transmission to target cells. These results provide insight into how microbiota directly affect HIV‐1 uptake thereby altering HIV‐1 susceptibility. Created in BioRender. Geijtenbeek, T. (2025) https://BioRender.com/i89c292.

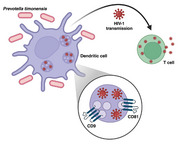

## 1 Introduction

Human immunodeficiency virus type 1 (HIV‐1) remains a serious global health problem, with the majority of newly acquired infections affecting young women in sub‐Saharan Africa [1, 2]. These women are 2.4 times more likely to acquire HIV‐1 than young male peers, the main route of HIV‐1 infection being via mucosal surfaces of the genital tract during sexual intercourse [1, 2]. Blocking mucosal transmission in the female genital tract is therefore key in preventing HIV‐1 infection in this vulnerable population [1, 2].

The composition of the vaginal microbiome residing in the female genital tract is important in susceptibility to HIV‐1 acquisition [3, 4, 5, 6, 7]. *Lactobacillus* spp. dominate a healthy vaginal microbiome and produce antimicrobial compounds providing a defence against pathogens [8, 9]. Susceptibility to HIV‐1 is enhanced when the microbial composition shifts from an optimal *Lactobacillus*‐dominated microbiome to a microbiome populated by bacterial vaginosis (BV)‐associated bacteria. The highest prevalence of BV is amongst females living in sub‐Saharan Africa [10]. It is therefore important to understand how BV affects HIV‐1 susceptibility. However, it remains unclear which mechanisms underly this enhanced susceptibility to HIV‐1 infection in the presence of BV‐associated bacteria, and the role of different bacterial species is currently unknown.

We have recently shown that *Prevotella timonensis*, an anaerobic bacterium associated with BV, enhanced susceptibility to HIV‐1 by affecting the antiviral function of Langerhans cells, a mucosal dendritic cell (DC) subset [11]. Langerhans cells reside in vaginal mucosal tissue, but the submucosa is also populated by other DC subsets, which may encounter *P. timonensis* and control the initial host immune response towards HIV‐1 [12, 13, 14]. However, it is unknown whether these DC subsets are affected by BV‐associated bacteria and whether this has an impact on altering HIV‐1 susceptibility.

Here, we have investigated the role of BV‐associated bacteria in altering HIV‐1 uptake, infection, and transmission in two different DC subsets present in vaginal mucosal tissue, monocyte‐derived DCs (moDCs) recruited upon inflammation [15, 16], and primary CD1c^+^ myeloid DCs present in steady‐state [14, 17]. Exposure to *P. timonensis*, but not to other BV‐associated bacteria, greatly enhanced viral uptake by moDCs and CD1c^+^ DCs into tetraspanin‐rich compartments. While *P. timonensis* did not induce infection of moDCs or CD1c^+^ DCs, the bacteria greatly enhanced HIV‐1 transmission to target cells by CD1c^+^ DCs but not by moDCs. These data suggest that DC subsets are differently affected by *P. timonensis*, and show that CD1c^+^ DCs enhance HIV‐1 susceptibility, whereas inflammatory moDCs prevent dissemination in the presence of *P. timonensis*. Our study underscores the importance of examining the role of the microbiome in viral pathogenesis to understand mucosal transmission and limit HIV‐1 infections.

## 2 Results

### 2.1 *Prevotella timonensis* Increases HIV‐1 Uptake in moDCs

**FIGURE 1:**
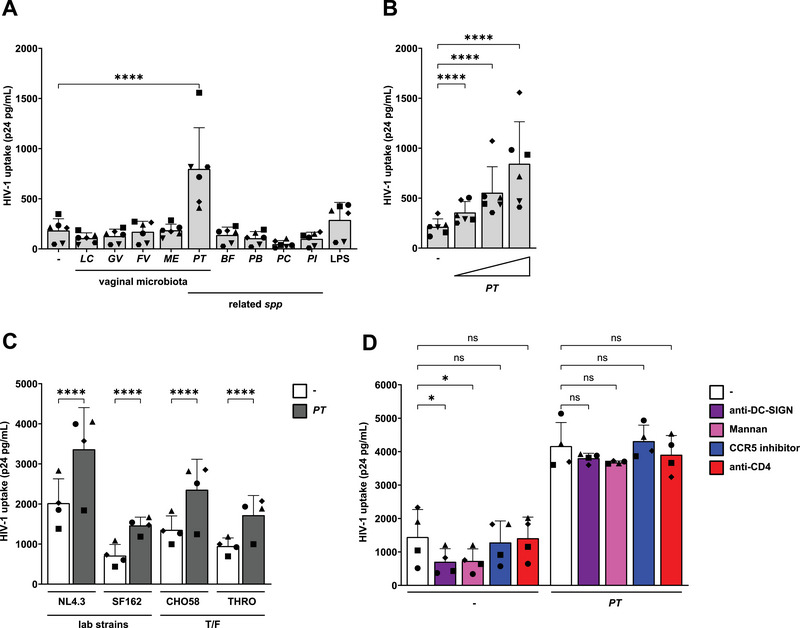
*Prevotella timonensis* increases HIV‐1 uptake in moDCs. MoDCs were stimulated with UV‐inactivated bacteria for 16 h (731 ng) and subsequently exposed to HIV‐1 (SF162; 7.3 ng) for 24 h unless otherwise specified. HIV‐1 uptake was quantified by p24 ELISA following cell lysis. (A) MoDCs were exposed to *Lactobacillus crispatus* (LC), *Gardnerella vaginalis* (GV), *Fannyhessea vaginae* (FV), *Megasphaera elsdenii* (ME), *Prevotella timonensis* (PT), *Bacteroides fragilis* (BF), *Prevotella bivia* (PB), *Prevotella copri* (PC), *Prevotella intermedia* (PI), or LPS derived from *Salmonella typhosa* (10 ng/mL). (B) MoDCs were stimulated with increasing amounts of *P. timonensis* (14.6, 146, 731 ng). (C) MoDCs were exposed for 6 h to 20 ng of lab‐adapted HIV‐1 strains NL4.3 (HEK293T‐produced) and SF162 (PBMC‐produced), and Transmitted Founder (T/F) variants CH058 and THRO. (D) MoDCs were pre‐treated with a DC‐SIGN blocking antibody (AZN‐D1, 20 µg/mL), CLR inhibitor Mannan (100 µg/mL), a CCR5 antagonist (Maraviroc, 30 µM), or a CD4 blocking antibody (RPA‐T4, 20 µg/mL) for 45 min prior to 6 h HIV‐1 exposure. Experiments were performed for four (C, D) to six (A, B) different donors measured in triplicate. Symbols represent independent donors, bars represent mean + SD. Statistical analysis was performed using a two‐way ANOVA with Tukey's multiple comparisons test. ****p < 0.0001, *p < 0.05.

To assess whether vaginal microbiota affect HIV‐1 uptake, moDCs were stimulated for 16 h with UV‐inactivated vaginal *L. crispatus* associated with a healthy microbiome, as well as *G. vaginalis*, *F. vaginae*, *M. elsdenii*, and *P. timonensis* bacteria associated with vaginal dysbiosis. Cells were subsequently exposed to HIV‐1 (SF162) for 24 h, and viral uptake was determined by p24 ELISA. *P. timonensis* but not the other bacteria increased HIV‐1 uptake (Figure 1A). Viral uptake was not enhanced by other *Prevotella* species, such as *P. bivia*, *P. copri*, and *P. intermedia*, or the related bacterium *Bacteroides fragilis*. We exposed moDCs to increasing amounts of *P. timonensis* and observed a concentration‐dependent increase in HIV‐1 uptake (Figure 1B).

Next, moDCs were incubated with different HIV‐1 strains, including CXCR4‐tropic strain NL4.3, CCR5‐tropic strain SF162, and CCR5‐tropic Transmitted Founder (T/F) variants, CH058 and THRO. In the presence of *P. timonensis*, viral uptake of all the different HIV‐1 strains was increased (Figure 1C), which suggests that this process is strain‐independent. Moreover, our data indicate that co‐receptors CCR5 and CXCR4 were not essential for this process, as enhanced uptake was observed for both R5‐ and X4‐tropic HIV‐1 strains. Consequently, we investigated whether known HIV‐1 (co‐)receptors [18, 19, 20, 21] were involved in enhanced HIV‐1 uptake induced by *P. timonensis*. Antibodies directed against DC‐SIGN and the general C‐type lectin receptor (CLR) inhibitor Mannan decreased HIV‐1 uptake by moDCs in the absence of *P. timonensis*, but none of the antibodies against DC‐SIGN and CD4 nor inhibitors for CLRs and CCR5 abrogated enhanced HIV‐1 uptake by *P. timonensis* (Figure 1D). These data strongly suggest that only *P. timonensis* bacteria, but not other vaginal bacteria tested, enhance HIV‐1 uptake of different viral strains by moDCs independent of known (co‐)receptors.

### 2.2 HIV‐1 Is Targeted to CD81‐ and CD9‐Positive Compartments in *P. timonensis*‐Exposed moDCs

**FIGURE 2:**
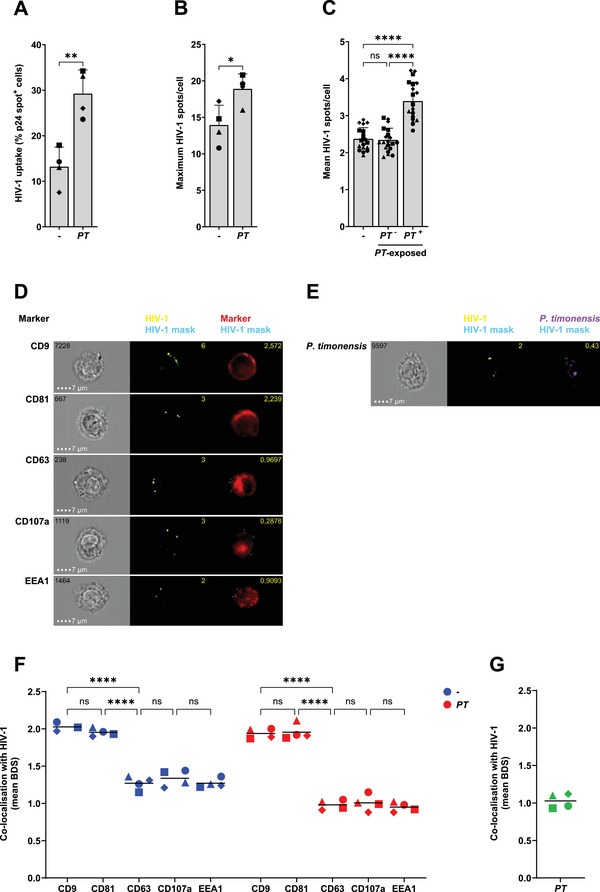
HIV‐1 localises to CD81‐ and CD9‐rich compartments, but does not co‐localise with *P. timonensis. P. timonensis* bacteria (CellTrace Violet (CTV)‐labelled (439 ng)) were incubated with HIV‐1 (SF162; 36.8 ng) for 1 h, and consequently incubated for 2 h with moDCs. Cells were fixed, stained, and acquired by imaging flow cytometry. DCs from each donor were exposed to HIV‐1 alone or together with *P. timonensis*. An average of 20,000 cells, positive for both HIV‐1 and the respective cellular marker, were analysed in each condition. A p24 spot count was performed using IDEAS software based on a mask created on the p24 image. (A) HIV‐1 uptake was determined by the percentage of cells positive for p24 spots. (B) Maximum number of p24 spots per cell. (C) Mean number of p24 spots per cell in untreated moDCs (‐), *P. timonensis*‐exposed moDCs with internalised *P. timonensis* (*PT* ^+^) and *P. timonensis*‐exposed moDCs with no internalised bacteria (*PT* ^−^). (D) Representative image of one cell per marker; respective brightfield image, overlay of p24 mask (blue) on p24 image (yellow) with spot count, and overlay of p24 mask (blue) on marker image (red) with Bright Detail Similarity (BDS) value. (E) Representative image of one cell; respective brightfield image, overlay of p24 mask (blue) on p24 image (yellow) with spot count, and overlay of p24 mask (blue) on *P. timonensis* image (purple) with BDS value. (F) Mean BDS values for HIV‐1 with CD9, CD81, CD63, CD107a or EEA1, determining co‐localisation. (G) Mean BDS value for HIV‐1 with *P. timonensis* bacteria. Experiments were performed for four different donors. Symbols represent independent donors, depicted with mean (F, G) or with bars representing mean + SD (A–C). Statistical analysis was performed using a paired two‐tailed *t*‐test (A, B) or a two‐way ANOVA with Tukey's multiple comparisons test (C, F). ****p < 0.0001, **p < 0.01, *p < 0.05.

To investigate the routing of HIV‐1 in moDCs exposed to *P. timonensis*, we applied imaging flow cytometry. After 2 h exposure to CTV labelled‐*P. timonensis* bacteria and HIV‐1, moDCs were analysed for p24 expression. Similar to those observed with HIV‐1 uptake by ELISA, *P. timonensis* increased the number of p24^+^ cells compared with untreated DCs (Figure 2A). *P. timonensis* exposed‐moDCs had an increased maximum number of HIV‐1 p24^+^ spots per cell compared with untreated moDCs (Figure 2B). We observed an increased average number of HIV‐1 p24^+^ spots in moDCs that had also internalised *P. timonensis*, compared with *P. timonensis*‐exposed but ‐negative moDCs (Figure 2C). Notably, the *P. timonensis*‐negative moDCs in the *P. timonensis*‐exposed condition had a similar number of HIV‐1 p24^+^ spots compared with untreated moDCs. We investigated the cellular localisation of these HIV‐1 p24^+^ spots by determining bright detail similarity (BDS) values. BDS is a method that can be used to determine the co‐localisation of intracellular HIV‐1 p24^+^ spots with cellular compartments by comparing the bright details of these two different stainings and determining the amount of overlap [22]. In untreated moDCs, HIV‐1 was internalised into compartments staining positive for tetraspanins CD9 and CD81 (Figure 2D), as has been previously observed [23]. The amount of co‐localisation was quantified and co‐localisation corresponded to a mean BDS of about 2 (Figure 2F). We did not observe co‐localisation with BDS values lower than 1.5. No co‐localisation was observed between HIV‐1 and early endosomal marker EEA1, late endosomal/multivesicular body (MVB) marker CD63 or lysosomal marker CD107a with a mean BDS value below 1,5. Notably, exposure to *P. timonensis* did not affect the internalisation pathway as HIV‐1 was also targeted to CD9‐ and CD81‐positive compartments and did not colocalise with endosomal or lysosomal compartments. Interestingly, the bacterium *P. timonensis* did not co‐localise with HIV‐1 (Figure 2E,G). We conclude that *P. timonensis* does not co‐localise with HIV‐1, even though its presence in moDCs is strongly associated with increased HIV‐1 uptake and enhanced internalisation into tetraspanin‐rich compartments.

### 2.3 Neither HIV‐1 Infection of nor Transmission by moDCs Is Affected by *P. timonensis*

**FIGURE 3:**
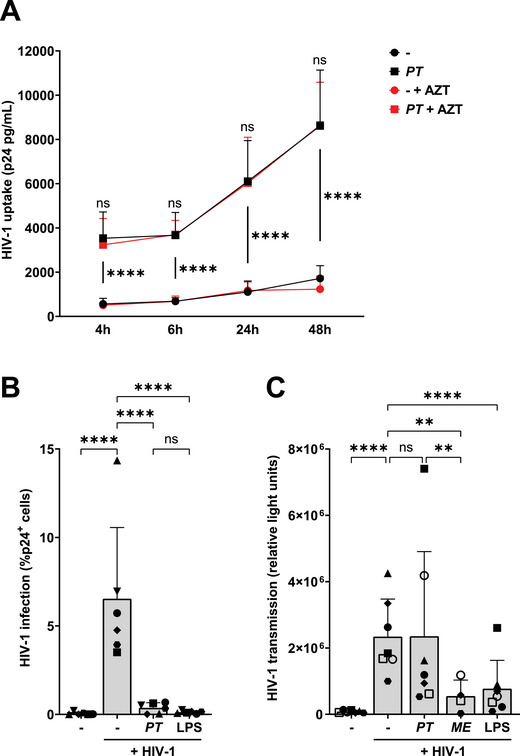
Enhanced *P. timonensis*‐induced viral uptake does neither lead to increased moDC infection nor transmission. MoDCs were stimulated with UV‐inactivated *Prevotella timonensis (PT)* for 16 h (731 ng) and subsequently to HIV‐1. (A) MoDCs were exposed to HIV‐1 (SF162; 36.8 ng) for 4 to 48 h in the presence or absence of replication inhibitor azidothymidine (AZT). HIV‐1 uptake was quantified by p24 ELISA following cell lysis. Symbols represent the mean of four independent donors measured in triplicate +SD. SD comparing US to *PT* and *PT* to *PT* + AZT are depicted. (B) HIV‐1 infection was assessed by flow cytometry after 5 days of HIV‐1 (MOI 0.015) exposure, following 16 h of PT or LPS (10 ng/mL) stimulation, by intracellular staining for the HIV‐1 capsid protein p24, and depicted as % p24^+^ cells. (C) MoDCs were stimulated with PT, *Megasphaera elsdenii (ME)*, or LPS for 16 h and subsequently exposed to HIV‐1 (SF162; 36.8 ng) for 6 h. Cells were harvested, extensively washed, and co‐cultured for 48 h with TZM‐bl target cells to determine viral transmission by luciferase reporter activity. Experiments were performed for six (B) or four to seven (C) different donors measured in triplicate. Symbols represent independent donors, bars represent mean + SD. Statistical analysis was performed using a two‐way ANOVA with Tukey's multiple comparisons test. ****p < 0.0001, **p < 0.01.

Localisation of HIV‐1 into tetraspanin‐rich compartments is associated with HIV‐1 preservation as well as HIV‐1 transmission [23, 24, 25, 26, 27]. We assessed the effect of *P. timonensis* on viral preservation and stability in moDCs over time after HIV‐1 exposure. HIV‐1 uptake was increased over time and not degraded in moDCs exposed to *P. timonensis*, suggesting that HIV‐1 is preserved for a longer period of time (Figure 3A). Blocking replication by the reverse transcriptase inhibitor azidothymidine (AZT) did not affect *P. timonensis‐*increased uptake at any time point, suggesting that replication is not involved in the increased HIV‐1 positivity. Next, we investigated infection of moDCs by exposure to HIV‐1 for an extended period of 5 days by determining intracellular p24 levels by flow cytometry. Despite the enhanced viral uptake in the presence of *P. timonensis*, HIV‐1 infection was decreased in *P. timonensis*‐exposed moDCs compared with untreated cells (Figure 3B; Figure S2). Viral infection was also decreased in LPS‐treated moDCs, in accordance with previous observations [28].

Certain DC subsets can transmit HIV‐1 to target cells independent of HIV‐1 infection [12, 19, 20, 29]. We investigated the effect of moDC exposure to *P. timonensis* on HIV‐1 transmission to susceptible target cells. TZM‐bl cells were used as a sensitive platform to quantify transmission. Notably, HIV‐1 transmission by *P. timonensis*‐exposed moDCs was comparable to that observed for untreated moDCs, whereas transmission was decreased by both *M. elsdenii*‐ and LPS‐treated moDCs (Figure 3C). Taken together, these data suggest that *P. timonensis* bacteria increase HIV‐1 uptake by moDCs, but do not enhance viral infection in moDCs nor increase viral transmission to target cells.

### 2.4 *Prevotella timonensis* Induces Comparable Cellular Activation and Type I IFN Responses in Primary Myeloid CD1c^+^ DCs and moDCs

**FIGURE 4:**
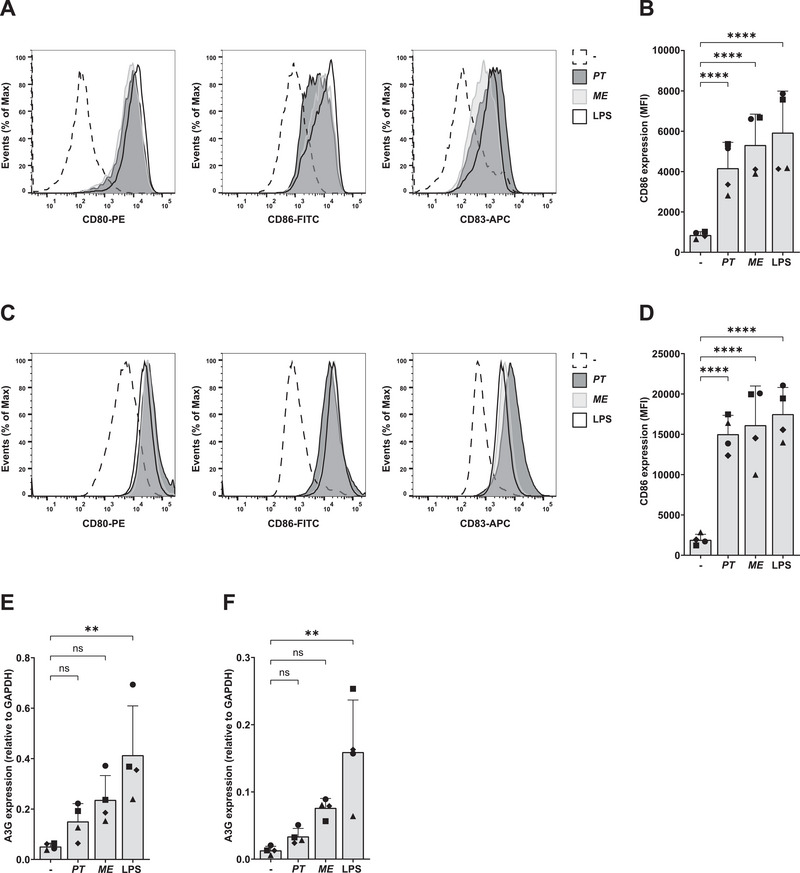
*P. timonensis* induces comparable cellular activation and type I IFN responses in both primary myeloid CD1c^+^ DCs and moDCs. Primary myeloid CD1c^+^ DCs (A, B, E) and moDCs (C, D, F) were stimulated for 16 h with UV‐inactivated *M. elsdenii (ME)* or *P. timonensis (PT)* (731 ng) or to LPS derived from *Salmonella typhosa* (10 ng/mL). (A–D) Surface expression was assessed by flow cytometry after staining for co‐stimulatory markers CD80 and CD86 and maturation marker CD83. Representative histograms of surface expression for one donor (A, C) or cumulative flow cytometry data for CD86 (B, D) expression by the geometric mean of the fluorescent intensity (MFI) are depicted. Symbols represent data of four independent donors measured in triplicate. (E, F). Expression of interferon‐stimulated‐gene APOBEC3G (A3G) was determined by quantitative real‐time PCR and normalised to household gene GAPDH. Symbols represent four independent donors, bars represent mean + SD. Statistical analysis was performed using a two‐way ANOVA with Tukey's multiple comparisons test. ****p < 0.0001, **p < 0.01.

MoDCs are observed in inflamed tissue; however, CD1c^+^ DCs are also present in the vaginal submucosa, where they have been shown to capture and transmit HIV‐1 [14, 30, 31]. We isolated the primary human CD1c^+^ DC subset from the blood by magnetic‐activated cell sorting and compared the cellular activation response following *P. timonensis* exposure to the response observed in moDCs. Exposure to *P. timonensis*, *M. elsdenii*, or LPS strongly induced expression of co‐stimulatory markers CD80 and CD86 as well as maturation marker CD83 on CD1c^+^ DCs as assessed by flow cytometry (Figure 4A,B; Figure S3). Similarly, exposure to vaginal *P. timonensis* enhanced surface expression of CD80, CD86, and CD83 on moDCs (Figure 4C,D; Figure S3), as previously observed with a *P. timonensis* isolate derived from a breast abscess [32]. *P. timonensis* exposure induced more CD83 but similar CD80 and CD86 expression on moDCs compared with *M. elsdenii* and LPS. Furthermore, CD1c^+^ DC exposure to *P. timonensis* did not induce expression of type I interferon‐stimulated genes (ISGs) APOBEC3G (A3G), ISG15, and TRIM5α compared with untreated CD1c^+^ DCs as measured by quantitative PCR (Figure 4E; Figure S3). Exposure to *M. elsdenii* showed a trend towards increased expression and LPS exposure induced strong expression of A3G. Moreover, in moDCs we also observed that LPS but not *P. timonensis*, induced strong upregulation of ISGs (Figure 4F; Figure S3). Exposure to either *P. timonensis* or *M. elsdenii* induced expression of pro‐inflammatory cytokines interleukin‐1 beta (IL‐1β) and IL‐6 in both CD1c^+^ DCs and moDCs (Figure S4), in line with previous observations [32]. Altogether, these data suggest that there is a comparable cellular activation and strong maturation in both DC subtypes following *P. timonensis* exposure without a type I interferon (IFN) response.

### 2.5 *Prevotella timonensis* Increases HIV‐1 Uptake into CD81/CD9‐Positive Compartments in CD1c^+^ DCs

**FIGURE 5:**
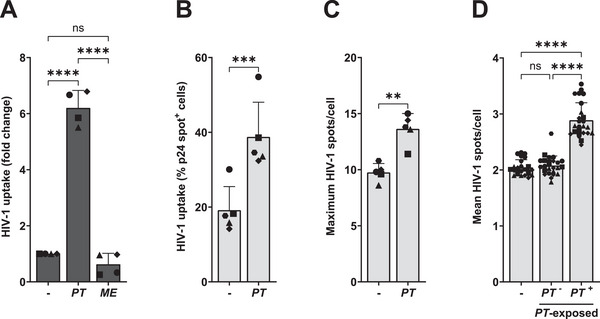
*P. timonensis* increases HIV‐1 uptake in CD1c^+^ DCs. (A) Primary myeloid CD1c^+^ DCs were stimulated with UV‐inactivated *Megasphaera elsdenii* (ME), or *Prevotella timonensis* (PT) for 16 h (731 ng) and subsequently exposed to HIV‐1 (SF162; 36.8 ng) for 24 h. HIV‐1 uptake was quantified by p24 ELISA following cell lysis and depicted as fold change compared with HIV‐1 uptake in moDCs not stimulated with microbiota. Symbols represent four independent donors measured in triplicate, bars represent mean +SD. (B–D) *P. timonensis* bacteria (CellTrace Violet (CTV)‐labelled (439 ng)) were incubated with HIV‐1 (SF162; 36.8 ng) for 1 h, and consequently incubated for 2 h with CD1c^+^ DCs. Cells were fixed, stained, and acquired by imaging flow cytometry. CD1c^+^ DCs from each donor were exposed to HIV‐1 alone or together with *P. timonensis*. An average of 44,000 cells, positive for both HIV‐1 and the cellular marker, were analysed per donor in both conditions. A p24 spot count was performed using IDEAS software based on a mask created on the p24 image. (B) HIV‐1 uptake determined by the percentage of cells positive for p24 spots. (C) Maximum number of p24 spots per cell. (D) Mean number of p24 spots per cell in untreated CD1c^+^ DCs (‐), *P. timonensis*‐exposed moDCs with internalised *P. timonensis* (*PT* ^+^), and *P. timonensis*‐exposed moDCs with no internalised bacteria (*PT* ^−^). Experiments were performed for five different donors. Symbols represent independent donors, bars represent mean + SD. Statistical analysis was performed using a two‐way ANOVA with Tukey's multiple comparisons test (A, D) or a paired two‐tailed *t*‐test (B, C). ****p < 0.0001, ***p < 0.001, **p < 0.01.

**FIGURE 6:**
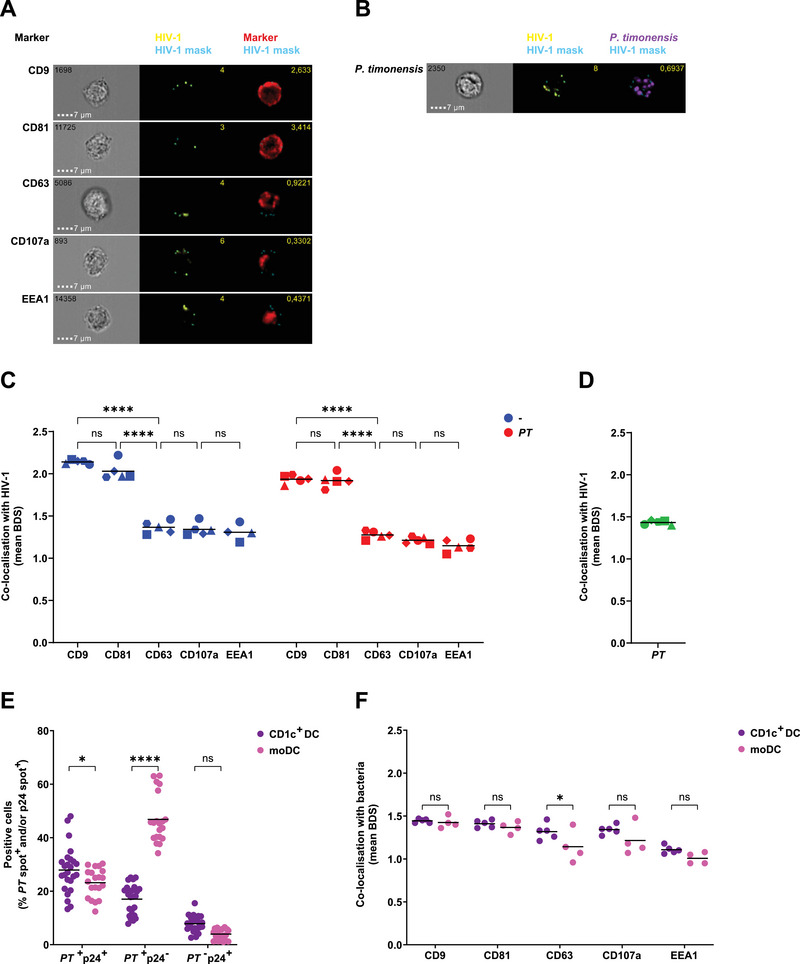
HIV‐1 localises to tetraspanin‐rich compartments in *P. timonensis*‐exposed CD1c^+^ DCs. *P. timonensis* bacteria (CellTrace Violet (CTV)‐labelled (439 ng)) were incubated with HIV‐1 (SF162; 36.8 ng) for 1 h, and consequently incubated for 2 h with CD1c^+^ DCs. Cells were fixed, stained, and acquired by imaging flow cytometry. CD1c^+^ DCs from each donor were exposed to HIV‐1 alone or together with *P. timonensis*. An average of 9000 cells, positive for both HIV‐1 and the respective cellular marker, were analysed in each condition. A p24 spot count was performed using IDEAS software based on a mask created on the p24 image. (A) Representative image of one cell per marker; respective brightfield image, overlay of p24 mask (blue) on p24 image (yellow) with spot count, and overlay of p24 mask (blue) on marker image (red) with bright detail similarity (BDS) value. (B) Representative image of one cell; respective brightfield image, overlay of p24 mask (blue) on p24 image (yellow) with spot count, and overlay of p24 mask (blue) on *P. timonensis* image (purple) with BDS value. (C) Mean BDS values for HIV‐1 with CD9, CD81, CD63, CD107a or EEA1, determining co‐localisation. (D) Mean BDS value for HIV‐1 with *P. timonensis* bacteria. (E, F) Based on a mask created on the *P. timonensis* image, a *P. timonensis* bacteria spot count was performed. (E) The percentage of moDCs and CD1c^+^ DCs positive for either *P. timonensis* spots (*PT* ^+^p24 ^−^) or p24 spots (*PT* ^−^p24 ^+^) or both (*PT* ^+^p24 ^+^) are depicted. (F) Mean BDS values for *P. timonensis* with CD9, CD81, CD63, CD107a, or EEA1, determining co‐localisation in moDCs and CD1c^+^ DCs. Experiments were performed for four (moDCs) or five (CD1c^+^ DCs) different donors. Symbols represent independent donors, depicted with mean. Statistical analysis was performed using a two‐way ANOVA with Tukey's (C) or Šídák's (E, F) multiple comparisons test. ****p < 0.0001, *p < 0.05.

We investigated the effect of vaginal microbiota on HIV‐1 uptake in primary CD1c^+^ DCs, by exposure to *P. timonensis* or *M. elsdenii* and p24 ELISA. Similar to the observations for moDCs, *P. timonensis* but not *M. elsdenii* strongly enhanced HIV‐1 uptake in CD1c^+^ DCs (Figure 5A). Next, we assessed HIV‐1 uptake in *P. timonensis*‐exposed CD1c^+^ DCs by imaging flow cytometry. We observed an increased number of p24^+^ cells in *P. timonensis‐*exposed CD1c^+^ DCs (Figure 5B) with an increased maximum number of HIV‐1 p24^+^ spots per cell (Figure 5C). In addition, CD1c^+^ DCs which have internalised *P. timonensis*, have more HIV‐1 p24^+^ spots per cell compared with *P. timonensis*‐exposed but ‐negative cells (Figure 5D). We investigated the cellular localisation of these HIV‐1 p24^+^ spots. In both untreated and *P. timonensis‐*exposed CD1c^+^ DCs, HIV‐1 localised to compartments staining positive for tetraspanins CD9 and CD81, but not to compartments positive for endosomal or lysosomal CD63, CD107a and EEA1 (Figure 6A,C). Similar to the moDCs, we also observed limited co‐localisation of HIV‐1 with *P. timonensis* in the CD1c^+^ DCs (Figure 6B,D). Interestingly, when comparing the total number of cells that have internalised *P. timonensis* or HIV‐1 alone or together, we observed that the majority of CD1c^+^ DCs have internalised both *P. timonensis* and HIV‐1, while in the moDC population the majority of cells were positive for *P. timonensis*, but not for HIV‐1 (Figure 6E). In addition, a larger number of moDCs had internalised *P. timonensis* compared with CD1c^+^ DCs. These data suggest that *P. timonensis* enhances HIV‐1 uptake more efficiently in CD1c^+^ DCs than in moDCs. The cellular localisation of *P. timonensis* was similar in both CD1c^+^ DCs and moDCs and there was no clear co‐localisation of *P. timonensis* with any of the markers for cellular compartments (Figure 6F). Taken together, *P. timonensis* strongly enhances HIV‐1 uptake in CD1c^+^ DCs into tetraspanin‐rich compartments.

### 2.6 *Prevotella timonensis* Increases HIV‐1 Transmission, but Not Infection in Primary CD1c^+^ DCs

**FIGURE 7:**
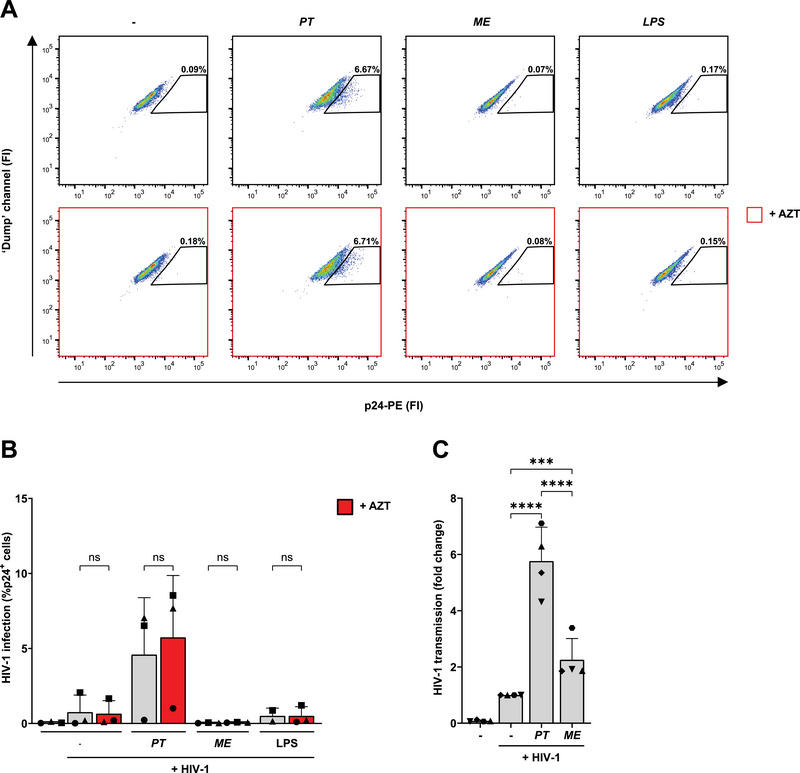
*P. timonensis* increases HIV‐1 transmission by CD1c^+^ DCs. Primary myeloid CD1c^+^ DCs were stimulated with UV‐inactivated *P. timonensis (PT), M. elsdenii (ME)* (731 ng) or to LPS (10 ng/mL) for 16 h and subsequently exposed to HIV‐1 (SF162; 36.8 ng). HIV‐1 infection was assessed by flow cytometry in the presence or absence of replication inhibitor AZT after 3 days of HIV‐1 exposure, following intracellular staining for the HIV‐1 capsid protein p24 (A, B). (A) Representative plots of different stimulations for one donor depicting p24 fluorescent intensity (FI) and percentage of HIV‐1^+^ cells. (B) HIV‐1 infection depicted as % p24^+^ cells. C. CD1c^+^ DCs were harvested 24 h post HIV‐1 exposure, extensively washed, and co‐cultured for 48 h with TZM‐bl target cells to determine viral transmission by luciferase reporter activity. HIV‐1 transmission to target cells was depicted as fold change compared with HIV‐1 transmission of cells not stimulated with microbiota. Experiments were performed for three (B) or four (C) different donors measured in triplicate. Symbols represent independent donors, bars represent mean + SD. Statistical analysis was performed using a two‐way ANOVA with Tukey's multiple comparisons test. ****p < 0.0001, ***p < 0.001.

We examined whether the enhanced viral uptake led to enhanced infection of CD1c^+^ DCs after overnight bacterial stimulation and exposure to HIV‐1 for 3 days with and without AZT. While we observed increased levels of p24 after *P. timonensis* exposure compared with untreated conditions, we observed no differences in p24 levels with or without the replication inhibitor AZT, suggesting the enhanced p24 levels were related to HIV‐1 uptake rather than productive infection in the CD1c^+^ DCs (Figure 7A,B). Next, we investigated the role of *P. timonensis*‐exposed CD1c^+^ DCs in HIV‐1 transmission to susceptible target cells. Strikingly, we observed an increased transmission after *P. timonensis* stimulation compared with untreated conditions (Figure 7C). Although *M. elsdenii* also enhanced transmission, the levels were lower than observed for *P. timonensis*. Overall, these data suggest that CD1c^+^ DCs show similar levels of HIV‐1 uptake and infection and a similar HIV‐1 localisation compared with moDCs following *P. timonensis* exposure. However, exposure to *P. timonensis* enhances HIV‐1 transmission to target cells by primary isolated CD1c^+^ DCs, but not by moDCs. These data demonstrate that different DC subsets which are present in the vaginal mucosa have distinct functions in BV‐associated HIV‐1 susceptibility.

## 3 Discussion

The composition of the vaginal microbiome in the female genital tract greatly influences susceptibility to HIV‐1 infection [3, 4, 5, 6, 7]. There is a high BV prevalence amongst females living in sub‐Saharan Africa [10], a population where the majority of newly acquired infections occur [1, 2]. *P. timonensis* bacteria are highly abundant in females with symptomatic and asymptomatic BV [33, 34]. Here, we investigated the role of BV‐associated bacteria in two functionally distinct DC subtypes that control immune responses but are also involved in HIV‐1 transmission: CD1c^+^ DCs present in vaginal tissue, and moDCs observed in inflamed tissues [12, 13, 14, 30, 31, 35]. We show that exposure to *P. timonensis* enhanced HIV‐1 uptake in both inflammatory moDCs and CD1c^+^ DCs and targeted the virus into tetraspanin‐rich compartments with distinct outcomes; *P. timonensis* enhanced HIV‐1 transmission by CD1c^+^ DCs but prevented dissemination by moDCs. Our data indicate that *P. timonensis* induces a general cellular mechanism leading to increased uptake of HIV‐1 into specific intracellular compartments with distinct consequences depending on the DC subset. These data and our previous work suggest that *P. timonensis* strongly impacts HIV‐1 susceptibility and that this might underlie the increased HIV‐1 susceptibility caused by BV.

MoDCs can originate from the differentiation of monocytes during inflammation and are thus observed in inflamed tissue [15, 16], whereas CD1c^+^ DCs are already present in the vaginal submucosa [14, 17]. Exposure of DCs to bacteria associated with a healthy microbiome, *L. crispatus*, or bacteria associated with and present in varying amounts in vaginal dysbiosis, *G. vaginalis*, *F. vaginae* and *M. elsdenii*, did not enhance HIV‐1 uptake. In both mucosal DC subsets, *P. timonensis* was the only BV‐associated bacterium which strongly enhanced HIV‐1 uptake. HIV‐1 uptake was neither enhanced by other *Prevotella* spp., *P. bivia*, *P. copri*, or *P. intermedia*, nor by a phylogenetically closely related *Bacteroides* spp., *B. fragilis*. Furthermore, we showed that in moDCs this *P. timonensis*‐enhanced uptake was independent of viral strains and known cellular HIV‐1 (co‐)receptors. We have recently shown that *P. timonensis* also enhances HIV‐1 uptake by primary LCs [11] and primary CD4^+^ T cells [36]. Our data therefore suggest that *P. timonensis*‐induction of HIV‐1 uptake is a general mechanism that leads to different outcomes.

BV‐associated bacteria induce cellular activation, thereby affecting DC function [32, 37], which might affect HIV‐1 interactions [26]. Exposure of both mucosal DC subsets to *P. timonensis* induced strong maturation of both DC subsets as well as expression of pro‐inflammatory cytokines. However, the other BV‐associated bacteria, such as *M. elsdenii*, similarly induced strong maturation and proinflammatory cytokines in both DC subsets. These data strongly suggest that the induction of HIV‐1 uptake by *P. timonensis* is independent of maturation as most BV‐associated bacteria, including LPS, induced maturation but did not enhance HIV‐1 uptake.

The increased uptake of HIV‐1 by *P. timonensis* was concentration‐dependent, as more bacteria induced more HIV‐1 uptake. With imaging flow cytometry, we followed the internalisation route of *P. timonensis* and HIV‐1. Both *P. timonensis* and HIV‐1 were internalised in moDCs as well as CD1c^+^ DCs. However, we did not observe co‐localisation of *P. timonensis* with HIV‐1 inside the cells. Notably, we observed that nearly all cells that were HIV‐1 positive, were also positive for *P. timonensis*, whereas *P. timonensis* binding was independent of HIV‐1 presence. These data suggest that *P. timonensis* actively affects either the cell, the virus, or both, thereby enhancing HIV‐1 uptake. Identifying which bacterial surface structures of *P. timonensis* are responsible for this increased viral uptake in multiple primary immune cells is important for designing targeted BV treatments. In addition, this might explain why bacterial species with a close phylogenetic relationship to *P. timonensis* do not increase uptake and may guide follow‐up studies investigating the potential HIV‐1‐enhancing abilities of a broader range of vaginal bacteria.

HIV‐1 uptake is mediated through various receptors and viral routing is dependent on both the specific receptor as well as the DC subset. In untreated DCs, HIV‐1 is internalised and targeted to a tetraspanin‐rich area [23]. Tetraspanin‐rich areas are suggested to be involved in transmission to susceptible target cells and protection of the virus against degradation [23, 24, 25, 26, 27]. By imaging flow cytometry, we observed a similar internalisation route of HIV‐1 in both moDCs and CD1c^+^ DCs under nonexposed conditions. Exposure to *P. timonensis* led to enhanced uptake and an increased number of intracellular HIV‐1 p24^+^ spots per cell in *P. timonensis*‐positive DCs. In both DC subsets, HIV‐1 was targeted to CD9‐ and CD81‐positive compartments in the cell periphery but not to early endosomes (EEA1), late endosomes/MVBs (CD63), or lysosomes (CD107a). HIV‐1 localisation to compartments containing CD81 and CD9 indicates the involvement of cholesterol‐mediated pathways [23, 26], which might be involved in *P. timonensis*‐enhanced HIV‐1 uptake. However, neither CD81 nor CD9 are directly involved in HIV‐1 binding and uptake, and therefore the underlying *P. timonensis*‐enhanced HIV‐1 uptake mechanism remains to be identified. Altogether, these data suggest that in *P. timonensis*‐exposed DCs HIV‐1 is targeted to compartments essential for viral transmission.

Strikingly, while *P. timonensis* enhanced viral internalisation by both DC subsets into tetraspanin‐positive compartments, only in CD1c^+^ DCs this led to enhanced HIV‐1 susceptibility by transmission to target cells. We did not observe an increase in transmission by moDCs. Moreover, the maturation of moDCs by LPS or *M. elsdenii* reduced transmission by moDCs. *P. timonensis* also activated moDCs but did not reduce transmission to target cells compared with untreated cells. It is therefore possible that the enhanced viral uptake induced by *P. timonensis* partially counteracts the decreased transmission due to cellular activation, resulting in a net similar transmission compared with nonexposed moDCs. Contrary to moDCs, maturation did not seem to hamper transmission in CD1c^+^ DCs. Exposure to *P. timonensis* and *M. elsdenii* induced cellular activation, and we observed a strong increase in transmission by *P. timonensis*‐exposed CD1c^+^ DCs and also a slight increase in transmission by *M. elsdenii*‐exposed CD1c^+^ DCs. HIV‐1 uptake was solely enhanced by *P. timonensis* but not by *M. elsdenii*, suggesting an additional role for maturation in transmission by CD1c^+^ DCs. In comparison, *P. timonensis* induced minor maturation of primary LCs but also strongly enhanced uptake and transmission by LCs [11], while the enhanced uptake in CD4^+^ T cells leads to enhanced productive infection [36]. Altogether, these studies suggest that although there might be a general mechanism underlying enhanced viral uptake induced by *P. timonensis* in multiple primary immune cells, this has different consequences on dissemination and HIV‐1 susceptibility depending on the additional effects on cellular activation of *P. timonensis* per cell type.

HIV‐1 targeting to tetraspanin‐rich areas in *P. timonensis*‐exposed DCs suggests protection of the virus and avoiding lysosomal degradation [23]. We observed the prolonged presence of p24 in both *P. timonensis*‐exposed moDCs and CD1c^+^ DCs as previously observed with LCs [11], suggesting that the virus is not routed into a degradation pathway in moDCs. Further research is needed to understand the differences in HIV‐1 transmission by moDCs and CD1c^+^ DCs after *P. timonensis* exposure.

*P. timonensis‐*enhanced uptake increased productive infection in CD4^+^ T cells [36], but did not increase infection in moDCs or CD1c^+^ DCs. Type I IFN responses are known to be antiviral and block HIV replication [38, 39, 40], as shown with LPS exposure. However, we did not observe any induction of type I IFN responses by *P. timonensis*‐exposed moDCs nor CD1c^+^ DCs, suggesting that type I IFN responses are not preventing HIV‐1 replication in these DC subsets. Both *P. timonensis* and LPS reduced infection in moDCs, consistent with earlier observations that DC maturation reduces productive infection of DCs [40, 41]. We did not observe productive infection of CD1c^+^ DCs after 3 days either in the presence or absence of *P. timonensis*. These data suggest that *P. timonensis* does not increase HIV‐1 replication in CD1c^+^ DCs and that the observed enhanced transmission is due to increased HIV‐1 uptake and retention but not due to increased viral replication. Indeed, *P. timonensis*‐increased HIV‐1 transmission by CD1c^+^ DCs was not inhibited by the antiretroviral replication inhibitor azidothymidine.

*P. timonensis* affects HIV‐1 uptake in different primary immune cells, raising the question of whether this enhanced HIV‐1 uptake and transmission could, besides in vaginal tissue, also play a role in other anatomical locations. In addition to labial and vaginal presence, *P. timonensis* has been identified in penile and oral swabs [33, 42]. Penile *Prevotella* colonisation is associated with an enhanced risk of HIV‐1 acquisition [42]. Furthermore, mucosal CD1c^+^ DCs found in the intestinal lamina propria respond to bacterial and viral TLR stimuli, and transmit HIV‐1 [43, 44, 45], altogether suggesting *P. timonensis* could also affect HIV‐1 susceptibility in other tissues and be relevant for infection of the male population. Our data strongly underscore the importance of microbiota in affecting HIV‐1 susceptibility and have uncovered a general mechanism induced by *P. timonensis* in enhancing HIV‐1 uptake by immune cells and in particular dendritic cells, which affects HIV‐1 susceptibility and dissemination.

### 3.1 Data Limitations and Perspectives

Here we focused on two different DC subsets present in vaginal tissue that are sensitive to HIV‐1 infection: moDCs recruited upon inflammation and CD1c^+^ DCs present in steady‐state. Investigating the differential effect of *P. timonensis* on transmission by both mucosal subsets will aid in understanding the role of specific immune cell subsets in BV‐associated HIV‐1 susceptibility. The mechanisms of increased uptake and internalisation observed in these mucosal subsets are important to further investigate to understand host‐virus interactions. Lastly, identifying which unique properties of *P. timonensis* are responsible for the general mechanism underlying enhanced HIV‐1 uptake observed across multiple primary cell types will be important for designing targeted BV treatments.

## 4 Materials and Methods

### 4.1 Antibodies and Reagents

The following anti‐human antibodies and reagents were used: p24‐PE (anti‐HIV‐1, 1:200, KC57‐RD1, Beckman Coulter), CD1c‐APC‐Cy7 (1:50, 331519, BioLegend), CD9‐APC (1:50, 312107, BioLegend), CD11c‐PE (1:25, 333149, BD Biosciences), CD63‐APC (1:500, 353007, BioLegend), CD80‐PE (1:12,5, 557227, BD Pharmingen), CD81‐APC (1:100, 349509, BioLegend), CD83‐APC (1:25, 551073, BD Pharmingen), CD86‐FITC (1:25, 555657, BD Pharmingen), CD107a‐APC (LAMP‐1, 1:500, 328619, BioLegend), EEA1‐AF647 (1:100, ab196186, Abcam), AZN‐D1 (anti‐DC‐SIGN, 20 µg/mL, produced in‐house), Mannan (100 µg/mL, Sigma‐Aldrich), RPA‐T4 (anti‐CD4, 20 µg/mL, BioLegend), LPS derived from *Salmonella typhosa* (10 ng/mL, Sigma), Ultracomp eBeads (01‐2222‐42, eBioscience)

HIV‐1 replication inhibitor azidothymidine (AZT, 20 µM) and CCR5 antagonist maraviroc (Mar, 30 µM) were obtained via the NIH AIDS Reagent Program NIAID. DCs were exposed to blocking antibodies or inhibitors for 45 min—1 h prior to HIV‐1 exposure.

### 4.2 Primary Cell Isolation and Cell Lines

CD14^+^ immature peripheral blood monocytes of healthy volunteer donors were isolated and differentiated to mature moDCs in 6 days, in the presence of GM‐CSF (800 U/mL) and IL‐4 (500 U/mL) as previously described [46, 47]. The human CD1c^+^ isolation kit for dendritic cells (Miltenyi Biotec) was used for the isolation of CD1c^+^ DCs from peripheral blood mononuclear cells (PBMCs) by magnetic labelling and separation according to the manufacturer's instructions. Purity was assessed by high expression of CD1c (APC‐Cy7‐conjugated) and CD11c (PE‐conjugated) via flow cytometry and cells were routinely >99% pure. MoDCs and CD1c^+^ DCs were cultured in Roswell Park Memorial Institute 1640 (RPMI1640, Gibco/Life Technologies), supplemented with fetal calf serum (FCS, 10%, Biological Industries), penicillin (10 U/mL, Gibco/Life Technologies), streptomycin (10 µg/mL Gibco/Life Technologies), and L‐glutamine (2 mM, Capricorn Scientific) at 37°C and 5% CO_2_. Four days after differentiation, moDCs were seeded at 1 × 10^5^/well in 100 µL in a 96‐well plate (Greiner), and cultured for 2 days. For DC maturation and IFN response assays, moDCs were seeded at 5 × 10^4^/well. MoDCs were exposed to microbiota on day 5, and after overnight incubation infected on day 6 as described below. CD1c^+^ DCs were seeded on the day of isolation (1 × 10^5^/well for virus assays, and 5 × 10^4^/well for maturation and IFN assays) and after a few hours of culturing, stimulated with microbiota as described below.

The TZM‐bl cell line [48] was maintained in Dubecco's modified Eagle's medium (Gibco Life Technologies), supplemented with FCS (10%), penicillin (10 U/mL), streptomycin (10 µg/mL), and L‐glutamine (2 mM) at 37°C and 10% CO_2_.

### 4.3 Vaginal Bacteria

*Bacteroides fragilis* (ATCC‐25285), *Fannyhessea vaginae* (DSMZ‐15829), *Gardnerella vaginalis* (DSMZ‐4944), *Lactobacillus crispatus* (DSMZ‐20584), *Megasphaera elsdenii* (DSMZ‐20460), *P. bivia* (DSMZ‐20514), *P. copri* (DSMZ‐18205), *P. intermedia* (DSMZ‐20706), and *P. timonensis* CRIS 5C‐B1 (alternative name *Hoylesella timonensis* [49], BEI Resources, HM‐136) bacteria were cultured as recommended by DSMZ (German Collection of Microorganisms and Cell Cultures GmbH). Bacteria were harvested during the logarithmic phase, washed extensively with phosphate‐buffered saline (PBS), and the optical density at 600 nm (OD_600 nm_) was measured. Culture purity was confirmed by Gram staining. All bacterial cultures were normalised to OD 1 in PBS and inactivated by exposure to five rounds of UV irradiation (100,000 µJ/cm^2^), using a UV Crosslinker (Stratagene). Complete loss of viability was verified in liquid culture. We determined the protein amount by BCA protein assay (Novagen) and this was used as the concentration for the different bacteria. MoDCs and CD1c^+^ DCs were exposed to 14.6–731 ng microbiota.

For imaging flow cytometry experiments, *P. timonensis* bacteria were fluorescently labelled with CellTrace Violet (CTV) Cell Proliferation Kit (ThermoFisher Scientific). Bacteria were incubated with 10 µM CTV for 20 min at RT while gently agitating and protected from light. To remove any free dye, PBS supplemented with 10% bovine serum albumin (BSA, Sigma) was added for 5 min and bacteria were pelleted and re‐suspended in PBS.

### 4.4 HIV‐1 Production

HIV‐1 SF162 was obtained from Dr. Jay Levy [50]. HIV‐1 SF162 viral stocks were generated using phytohemagglutinin (PHA)‐stimulated PBMCs from human origin. HIV‐1 T/F viruses CH058 and THRO were produced as previously described [51]. HIV‐1 NL4.3 was produced by transfection of human embryonic kidney 293T cells with proviral NL4.3 plasmids (10 µg), obtained via the NIH AIDS Reagent Program NIAID. Transfection was performed using GeneJuice (Novagen) transfection reagent according to the manufacturer's instructions. Viruses were harvested on day 2 and filtered over a 0.2 µm pore‐size filter (Fisher Scientific). TCID50 of produced HIV‐1 viral stocks was determined by the indicator cell line TZM‐bl, and the amount of HIV‐1 capsid protein p24 quantified by p24 antigen enzyme‐linked immunosorbent assay (ELISA, ZeptoMetrix)

### 4.5 DC Maturation, Interferon Response, and mRNA Analyses

MoDCs and CD1c^+^ DCs were exposed to vaginal microbiota or LPS for 16 h. To quantify surface expression of maturation markers CD80, CD83 and CD86, cells were washed and staining was performed in TSM buffer (TSM (20 mM Tris, 150 mM NaCl, 1 mM CaCl2, 2 mM MgCl2) at pH 7.4) supplemented with 0.5% BSA for 30 min at 4°C. Cells were measured by flow cytometry using FACS Canto II (BD Biosciences) and Ultracomp eBeads were used to identify spectral overlap. Data analysis was performed using FlowJo v10.10.0 software (TreeStar) and the guidelines for the use of flow cytometry and cell sorting in immunological studies were adhered to [52]. Live cells were gated using FSC and SSC, and the geometric mean of the fluorescent intensity (MFI) of surface markers CD80, CD83 and CD86 was determined.

For mRNA analyses of ISGs and cytokines, cells were lysed and mRNA was extracted using the mRNA Catcher PLUS purification kit (ThermoFisher Scientific). Subsequently, mRNA was transcribed to cDNA using a reverse transcriptase kit (Promega). Quantitative PCR amplification was performed in the presence of SYBR green (ThermoFisher Scientific) using the 7500 Fast Real‐Time PCR System (Applied Biosciences). Cycling threshold (Ct) values were obtained, and expression mRNA levels from the target gene of interest were normalised to the expression mRNA levels from the household gene GAPDH, according to the formula: N_t_ = 2^Ct(GAPDH)‐Ct(target)^. Primers were designed with Primer Express Software v2.0 (Applied Biosciences), and the following primers were used:

APOBEC3G: Fw_TTGAGCCTTGGAATAATCTGCC; Rv_TCGAGTGTCTGAGAATCTCCCC;

GAPDH: Fw_CCATGTTCGTCATGGGTGTG; Rv_GGTGCTAAGCAGTTGGTGGTG;

ISG15: Fw_TTTGCCAGTACAGGAGCTTGTG; Rv_GGGTGATCTGCGCCTTCA;

TRIM5α: Fw_AGAACATACGGCCTAATCGGC; Rv_CAACTTGACCTCCCTGAGCTTC;

IL‐1β: Fw_TTTGAGTCTGCCCAGTTCCC; Rv_TCAGTTATATCCTGGCCGCC;

IL‐6: Fw_TGCAATAACCACCCCTGACC; Rv_TGCGCAGAATGAGATGAGTTG.

### 4.6 HIV‐1 Uptake and Infection Assays

Following overnight bacterial exposure, DCs were pretreated with AZT for 1 h and consequently exposed to HIV‐1 (SF162; 7.3–36.8 ng) for 4–48 h. After HIV‐1 exposure, cells were extensively washed to remove unbound virus, lysed, and viral uptake was quantified by RETRO‐TEK HIV‐1 p24 ELISA (pg/mL), according to the manufacturer's instructions (ZeptoMetrix). For comparison of viral uptake between viruses, cells were exposed to 20 ng of SF162, NL4.3, CH058, and THRO for 6 h.

For infection assays, DCs were cultured in the absence of AZT, exposed to SF162 (MOI 0.015), and fixed using 4% (w/v) paraformaldehyde (PFA) after 3 to 5 days of infection. As a control for productive infection, cells were exposed to AZT. For intracellular p24 staining, cells were permeabilised for 10 min after fixation, using PBS supplemented with 0.5% saponin (Sigma) and 0.5% BSA. Cells were analysed by flow cytometry, live cells were gated using FSC and SSC, and the amount of HIV‐1 infection per donor was determined by the % p24^+^ cells.

### 4.7 HIV‐1 Transmission Assays

For transmission assays, the adherent target cell line TZM‐bl was seeded (1 × 10^4^/well in 100 µl) in a 96‐well plate and left to adhere for 24 h. MoDCs and CD1c^+^ DCs were exposed to microbiota overnight, and subsequently to HIV‐1 (SF162; 36.8 ng) in the absence or presence of AZT. After 6 and 24 h of HIV‐1 exposure, respectively, cells were harvested, extensively washed to remove unbound virus, transferred to target TZM‐bl cells, and co‐cultured for 48 h. After 48 h, DCs were washed away and HIV‐1 production in TZM‐bl was assessed by measuring luciferase activity (relative light units (R.L.U.)) by BriteLite plus reporter gene assay according to manufacturer's instructions (Perkin Elmer).

### 4.8 Imaging Flow Cytometry

For imaging flow cytometry experiments, CTV labelled‐*P. timonensis* bacteria were incubated with HIV‐1 (SF162; 36.8 ng) for 1 h, and subsequently incubated for 2 h with DCs in the presence of AZT. As a control, DCs were also exposed to HIV‐1 or *P. timonensis* alone. After 2 h, DCs were fixed, permeabilised and stained intracellular for p24 and CD9, CD63, CD81, CD107a, or EEA1. Cells were measured at 60× magnification by imaging flow cytometry (Amnis ImageStream^X^ Mark II, Cytek Biosciences) and analysis was performed using IDEAS v6.2 software (Amnis). Cells were gated as follows: single cells (aspect ratio vs. area), cells in focus (normalised frequency vs. gradient RMS), cells with marker (Intensity SSC vs. Intensity marker), cells with expression of marker and exclusion of saturated pixels (Max Pixel vs. Intensity marker) and p24 positive cells (Max Pixel vs. Intensity p24; Figure S1). In *P. timonensis*‐exposed cells, in addition, *P. timonensis* positive cells (Max Pixel vs. Intensity *P. timonensis*) were determined. Per condition 1–5 × 10^5^ cells positive for both the marker and HIV‐1 were analysed. For each fluorescent channel, samples stained for a single marker were acquired to determine spectral overlap. To perform a p24 spot count, a mask was created on the p24 image by using respectively spot, peak and range restrictions (Figure S1). Similarly, a mask on the *P. timonensis* image was created to perform a *P. timonensis* spot count (Figure S1). Quantitative analysis of co‐localisation of p24 with *P. timonensis*, CD9, CD63, CD81, CD107a and EEA1 was determined by mean BDS values of p24 spots with the respective marker. Furthermore, BDS was used to determine co‐localisation of *P. timonensis* spots with these cellular markers.

### 4.9 Data Analysis and Statistics

Generation of graphs and statistical analyses were performed using GraphPad Prism v10.2.0 software (GraphPad Software Inc.). A paired two‐tailed *t*‐test was performed for pairwise comparisons. A two‐way ANOVA test was performed for multiple comparisons of unpaired grouped data between donors with a Šídák's or Tukey's multiple comparisons test. Results are presented as mean + SD. Statistical significance was set at *p* < 0.05 (ns = not significant, *p < 0.05; **p < 0.01, ***p < 0.001, ****p < 0.0001).

## Author Contributions

Marleen Y. van Smoorenburg designed experiments. Marleen Y. van Smoorenburg, John L. van Hamme, and Ester B. M. Remmerswaal performed the experiments. Celia Segui‐Perez and Karin Strijbis contributed essential research materials and scientific input. Marleen Y. van Smoorenburg, Ester B. M. Remmerswaal, and Teunis B. H. Geijtenbeek analysed and interpreted data. Marleen Y. van Smoorenburg and Teunis B. H. Geijtenbeek wrote the manuscript with input from all listed authors. Teunis B. H. Geijtenbeek supervised all aspects of the study.

## Ethics Statement

This study was performed in accordance with the ethical principles set out in the Declaration of Helsinki, and approved by the Medical Research Ethics Committee (MREC) of the Amsterdam University Medical Centres (UMC) and the Ethics Advisory Board of Sanquin Blood Supply Foundation (Amsterdam, the Netherlands). According to the Medical Research Involving Human Subjects Act (WMO) and MREC of the Amsterdam UMC, the use of buffy coats is not subjected to informed consent. Human buffy coats were obtained from healthy volunteers after blood donation at Sanquin. All buffy coat samples were handled anonymously and in accordance with relevant guidelines and regulations as set out in the Amsterdam UMC Research Code.

## Conflicts of Interest

The authors declare no conflicts of interest.

### Peer Review

The peer review history for this article is available at https://publons.com/publon/10.1002/eji.202451192

## Supporting information



Supporting Information

## Data Availability

The data are available from the corresponding author upon reasonable request.
