# Supplementary material for: Vaginal Prevotella timonensis Bacteria Enhance HIV‐1 Uptake and Differentially Affect Transmission by Distinct Primary Dendritic Cell Subsets
Source: Eur J Immunol. 2025 Mar 12;55(3):e202451192. doi: 10.1002/eji.202451192 (PMC11898549; doi:10.1002/eji.202451192)
Supplement: Supplementary file 1 — Supporting Information [file EJI-55-e202451192-s001.pdf]

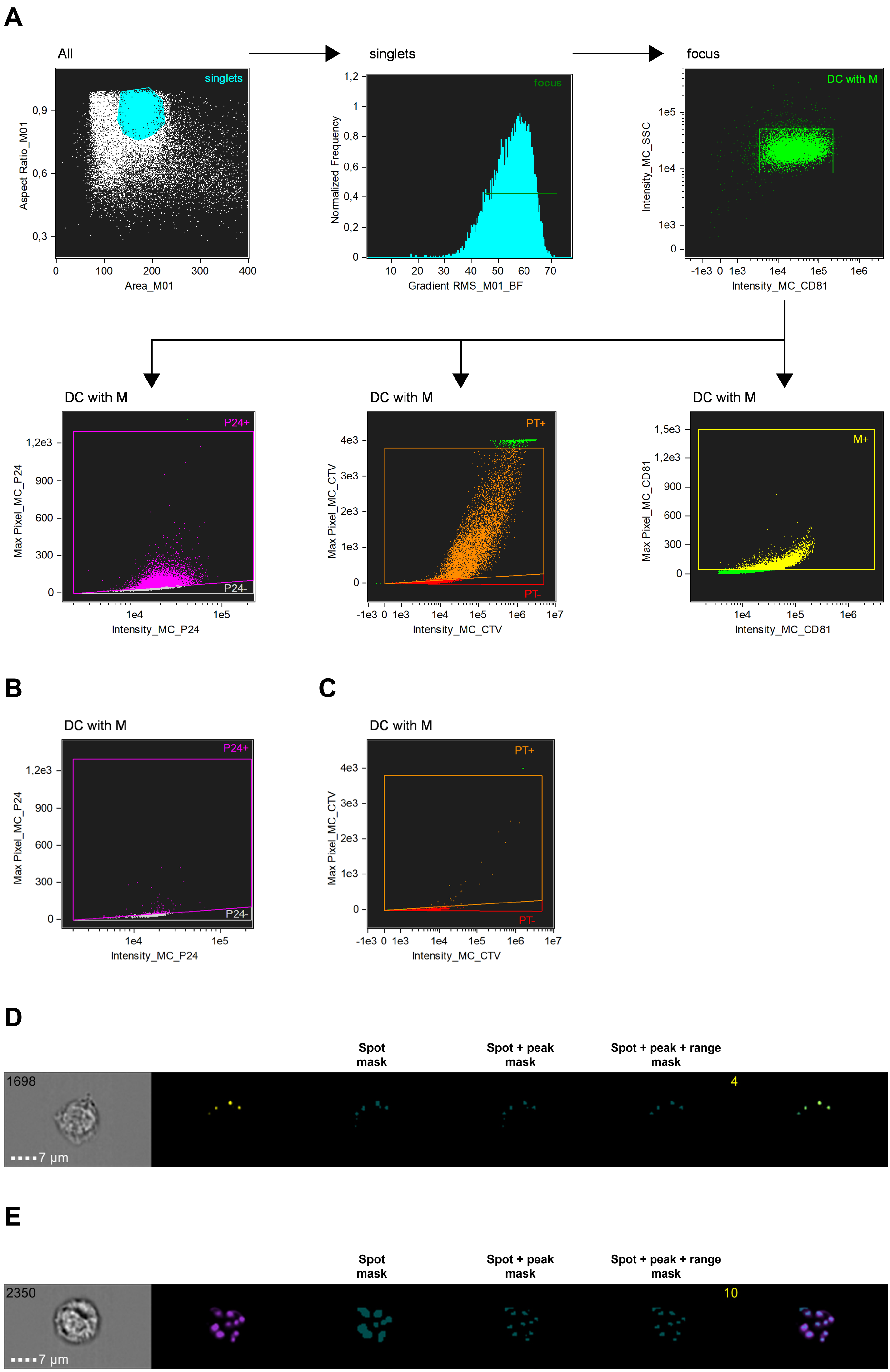
A
All
singlets
Aspect Ratio_M01
Area_M01
0,9
0,6
0,3
0
100
200
300
400
singlets
focus
Normalized Frequency
Gradient RMS_M01_BF
1,2
1
0,8
0,6
0,4
0,2
0
10
20
30
40
50
60
70
focus
DC with M
Intensity_MC_SSC
Intensity_MC_CD81
1e5
1e4
1e3
0
-1e3
0
1e3
1e4
1e5
1e6
DC with M
P24+
P24-
Max Pixel_MC_P24
Intensity_MC_P24
1,2e3
900
600
300
0
1e4
1e5
DC with M
PT+
PT-
Max Pixel_MC_CTV
Intensity_MC_CTV
4e3
3e3
2e3
1e3
0
-1e3
0
1e3
1e4
1e5
1e6
1e7
DC with M
M+
Max Pixel_MC_CD81
Intensity_MC_CD81
1,5e3
1,2e3
900
600
300
0
1e4
1e5
1e6
B
DC with M
P24+
P24-
Max Pixel_MC_P24
Intensity_MC_P24
1,2e3
900
600
300
0
1e4
1e5
C
DC with M
PT+
PT-
Max Pixel_MC_CTV
Intensity_MC_CTV
4e3
3e3
2e3
1e3
0
-1e3
0
1e3
1e4
1e5
1e6
1e7
D
Spot mask
Spot + peak mask
Spot + peak + range mask
1698
4
7 µm
E
Spot mask
Spot + peak mask
Spot + peak + range mask
2350
10
7 µm

Figure S1. Gating strategy and mask creation for imaging flow cytometry.

Experiments were performed for moDCs and CD1c^{+} DCs, and a similar gating strategy was performed for both cell types. **A.** An example of the gating strategy in CD1c^{+} DCs exposed to *P. timonensis* and HIV-1 in a CD81-stained sample. Single cells were gated based on the Area versus the Aspect Ratio in the Brightfield channel. Next, cells in focus of the flow system were gated based on Gradient RMS in the Brightfield channel. Next, cells expressing the respective marker (DC with M) were gated based on Intensity of the marker versus SSC Intensity. Consequently, to exclude cells not expressing the marker, cells positive for the marker (M+) were gated based on Intensity of the marker versus Max Pixel of the marker. Furthermore, HIV-1^{+} cells (p24+) were gated based on Intensity of p24 versus Max Pixel of p24, and in *P. timonensis*-exposed cells *P. timonensis*+ cells (*PT+*) were gated based on Intensity of *P. timonensis* versus Max Pixel of *P. timonensis*. **B.** An example of the p24+ gate in CD1c^{+} DCs exposed to *P. timonensis* but not to HIV-1 in a sample stained for CD81 and p24. **C.** An example of the *P. timonensis*+ gate in CD1c^{+} DCs exposed to HIV-1 but not to *P. timonensis* in a sample stained for CD81 and p24. **D.** Representative image of one cell showing build-up of the p24 mask; brightfield image, p24 image, p24 mask with spot restriction, p24 mask with spot and peak restriction, p24 mask with spot, peak and range restriction and spot count, overlay final p24 mask on p24 image. **E.** Representative image of one cell showing build-up of the *P. timonensis* mask; brightfield image, *P. timonensis* image, *P. timonensis* mask with spot restriction, *P. timonensis* mask with spot and peak restriction, *P. timonensis* mask with spot, peak and range restriction and spot count, overlay final *P. timonensis* mask on *P. timonensis* image.

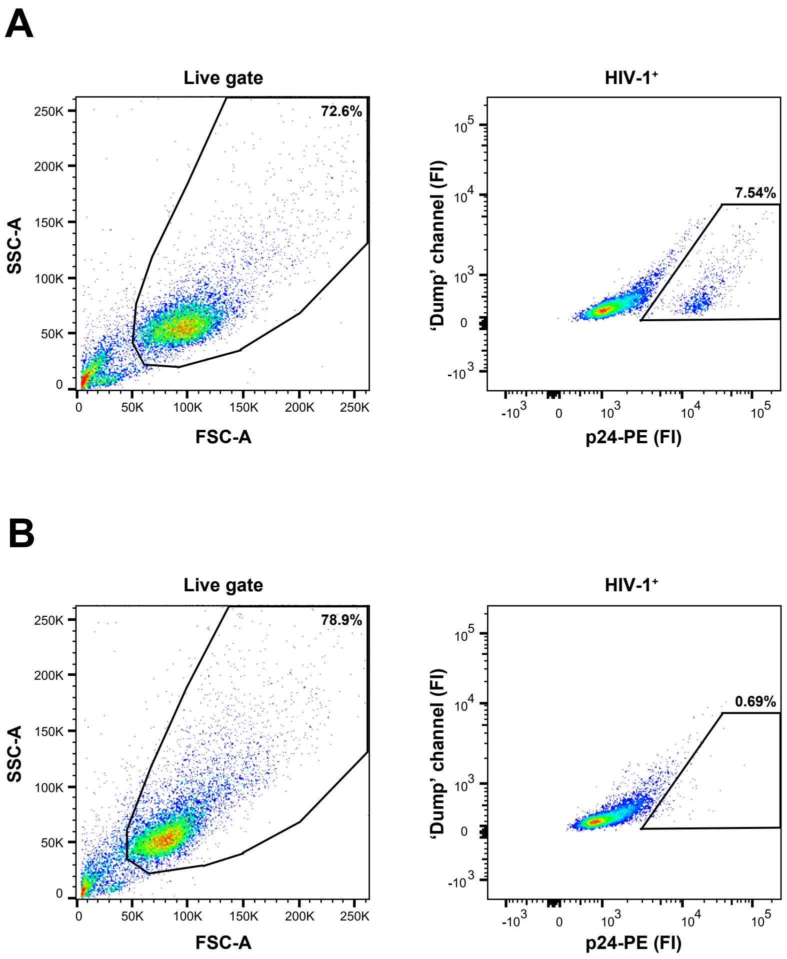

Figure S2. *P. timonensis* exposure decreases HIV-1 infection in moDCs.

MoDCs were left untreated (**A**) or stimulated with UV-inactivated *Prevotella timonensis (PT)* for 16 h (731 ng) (**B**). HIV-1 infection was assessed by flow cytometry after five days of HIV-1 (MOI 0.015) exposure, following intracellular staining for the HIV-1 capsid protein p24. Representative example of gating for live cells and HIV-1^{+} cells by p24 detection, fluorescence intensity (FI).

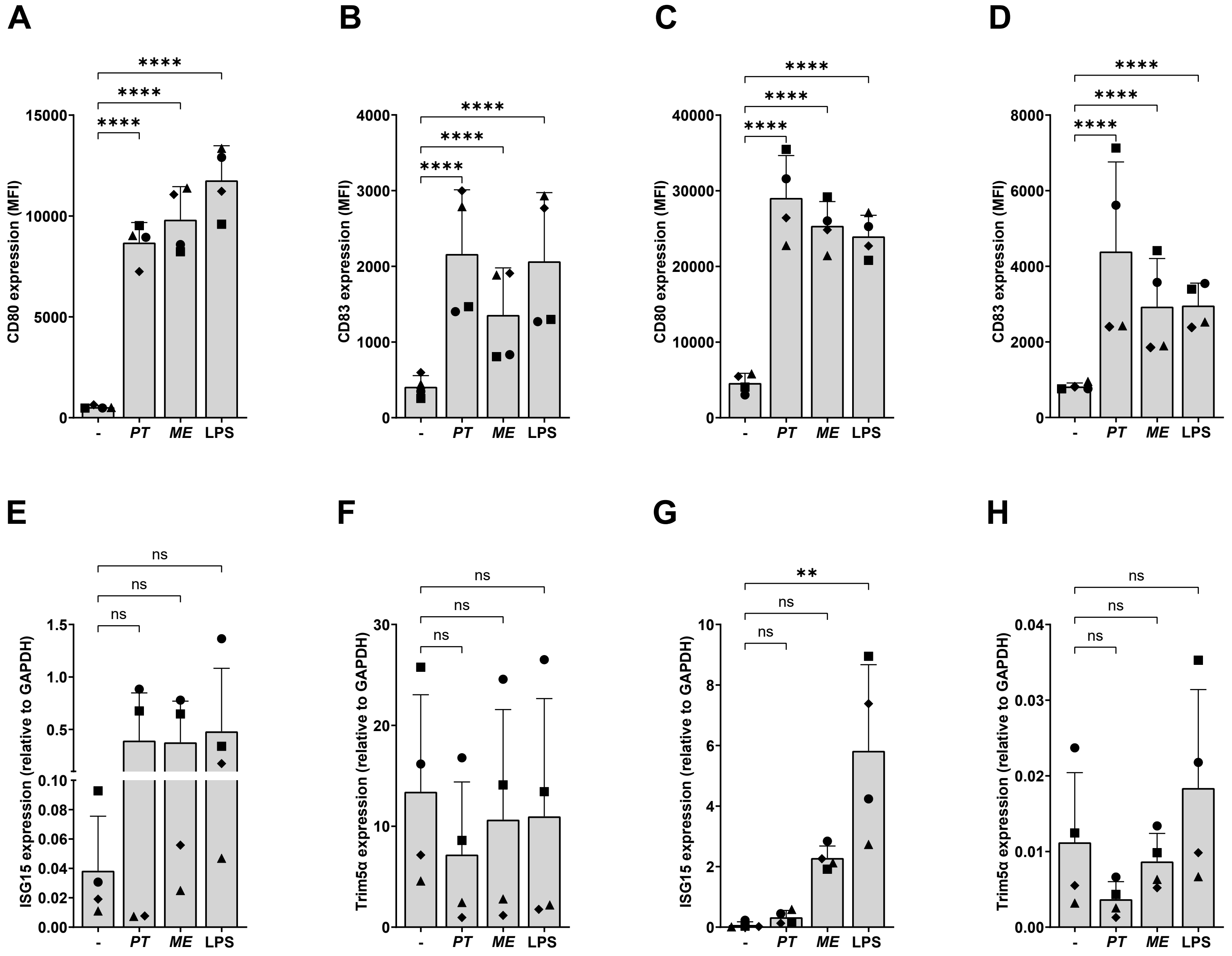

Figure S3. ***P. timonensis*** **induces comparable cellular activation and type I IFN responses in primary myeloid CD1c⁺ DCs and moDCs.**

Primary myeloid CD1c⁺ DCs (**A, B, E, F**) and moDCs (**C, D, G, H**) were stimulated for 16 h with UV-inactivated *P. timonensis (PT)*, *M. elsdenii (ME)* (731 ng) or LPS (10 ng/mL). **A-G**. Surface expression was assessed by flow cytometry and cumulative data of CD80 (**A, C**) and CD83 (**B, D**) expression by geometric mean of the fluorescent intensity (MFI) are depicted. Symbols represent data of four independent donors measured in triplicate. **E-H**. Expression of interferon-stimulated gene 15 (ISG15) (**E, G**) and TRIM5α (**F, H**) determined by quantitative real-time PCR and normalised to household gene GAPDH. Symbols represent four independent donors, bars represent mean +SD. Statistical analysis was performed using a two-way ANOVA with Tukey's multiple comparisons test. **** $P < 0.0001$, ** $P < 0.01$.

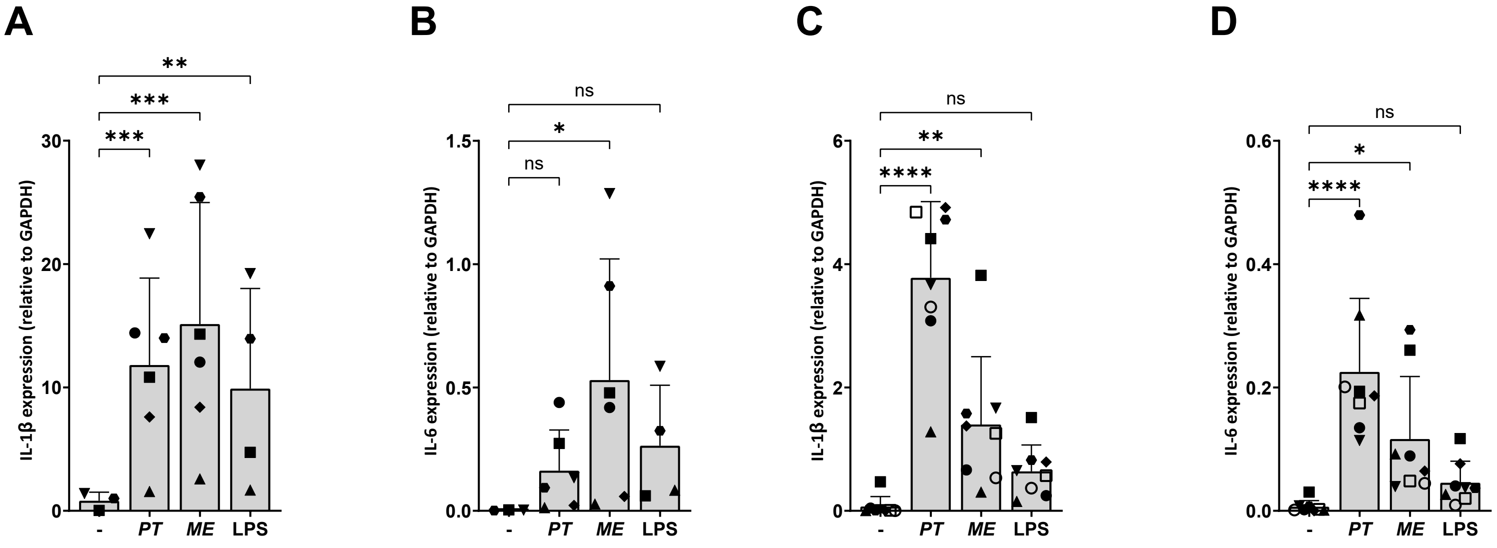

Figure S4. Cytokine production is induced upon cellular activation in primary myeloid CD1c$^+$ DCs and moDCs.

Primary myeloid CD1c$^+$ DCs (**A, B**) and moDCs (**C, D**) were stimulated for 16 h with UV-inactivated *P. timonensis (PT)*, *M. elsdenii (ME)* (731 ng) or LPS (10 ng/mL). Expression of interleukin-1 beta (IL-1β) (**A, C**) and IL-6 (**B, D**) determined by quantitative real-time PCR and normalised to household gene GAPDH. Symbols represent six (**A, B**) to eight (**C, D**) independent donors, bars represent mean +SD. Statistical analysis was performed using a two-way ANOVA with Tukey's multiple comparisons test. **** $P < 0.0001$, *** $P < 0.001$, ** $P < 0.01$, * $P < 0.05$.
